# Therapy Management of Metabolic Disorder Comorbidity With Depression

**DOI:** 10.3389/fpsyg.2021.683320

**Published:** 2021-08-02

**Authors:** Hua Luo, Zheng-Li Jiang, Yu Ren

**Affiliations:** ^1^Department of Orthopedics, Taizhou Hospital of Zhejiang Province Affiliated to Wenzhou Medical University, Taizhou, China; ^2^Department of Pharmacy, Taizhou Hospital of Zhejiang Province Affiliated to Wenzhou Medical University, Taizhou, China

**Keywords:** metabolic disorder, comorbidity, depression, medication, therapy management

## Abstract

Depression is a common disease that seriously endangers the physical and mental health of human beings, and it often coexists with other metabolic disorders such as diabetes and cancer. There have been endless reports on the mechanism, prevention, and cure of comorbidity because of its high incidence and poor prognosis and the increased burden on the family and society. There may be a specific comorbid basis and causal relationship between depression and metabolic diseases. Depression in patients with metabolic disorders can be effectively alleviated through psychotherapy and medication. The timely and effective treatment of depression can significantly improve the quality of life of patients with metabolic disorders, reduce their psychological burden, and promote the effective treatment of metabolic diseases. This study reorganized the research progress on the management of metabolic disorder comorbidity with depression.

## Introduction

Depression is a mood disorder that is clinically characterized by a significant and persistent feeling of sadness and loss of interest or pleasure, which leads to impaired social interaction, such as decreased communication, the diminished ability of cooperation, and black mood (Kupferberg et al., 2016). It has a high rate of suicide and disability (Li et al., 2017). More than 450 million people worldwide suffer from mental or behavioral disorders, and nearly 1 million commit suicide each year (Chisholm, 2015).

Metabolic disorders are metabolic syndromes that integrate physiological, biochemical, and clinical factors mainly manifested as insulin resistance, hyperglycemia, visceral fat accumulation, dyslipidemia, endothelial dysfunction, elevated blood pressure, etc. For the past few years, it has been found that the rate of metabolic diseases comorbidity with depression has increased significantly, and the prevalence of severe depression in patients with hypertension and myocardial infarction is 29 and 22%, respectively (Chisholm, 2015). Patients with depression have a 41% increased risk of diabetes and a 32% increased risk of type 2 diabetes (Yu et al., 2015), and their complex diseases can increase the prevalence and mortality of cardiovascular disease, diabetes, fatty liver disease (Saklayen, 2018). Recent studies have shown that diabetics have a higher risk of depression, epidemiological studies show that about 30% of diabetic patients have depressive symptoms, of which 10% are severely depressive (Bot et al., 2012). Also, depression was speculated to aggravate the abnormal metabolism in patients with a metabolic disorder, increasing the risks of cardiovascular complications (Katon et al., 2004; Zhang et al., 2005).

However, at present, the specific mechanism for the occurrence and development of metabolic disease comorbidity with depression is still unclear. It mainly focuses on the hypothalamic-pituitary-adrenal (HPA) axis, oxidative stress, immune system, glycolipid metabolism disorder, vascular endothelial function, and other aspects, which leads to the two kinds of diseases a certain extent mutually cause and effect (Dean and Keshavan, 2017). Professor Tao insisted that depression is a metabolic disorder with clinical characteristics of high uric acid, high homocysteine, high histamine, low serotonin, low dopamine, and low norepinephrine. The imbalance of the above substances leads to the damage of brain tissue and nerve cells, which leads to a series of mental and psychological symptoms, such as significant and lasting depression, reduced interest, decreased or slow speech behavior, gastrointestinal disorders, food allergies, sensitive and paranoid, the pursuit of pathological perfection. Long-term gastrointestinal dysfunction (intestinal micro ecological imbalance, intestinal mucosa damage, and intestinal immune function decline) prone to cause food allergy, further aggravate the decline of digestion and absorption function, the lack of nutrients, and in turn aggravate metabolic abnormalities. Inflammatory mediators produced by allergies enter the brain through the blood and induce the inflammatory response of brain nerve cells, aggravating mental symptoms. Also, mood disorders can directly cause immune dysfunction. Lack of diet control, poor treatment compliance, poor glucose/blood lipid/blood pressure control may contribute to an increased incidence of metabolic diseases in patients with depression (Tao and Li, 2015). The comorbidities of metabolic diseases with depression lead to poorer clinical outcomes. The possibility of patients with metabolic disorders comorbidity with depression is twice higher than the healthy people. For the existence of another disease, leading to two kinds of disease prognosis getting worse, including the severity of the disease, treatment resistance and case fatality rate increases, the cost of treatment increased obviously, the quality of life and self-management ability, even shortening life expectancy (Snoek et al., 2015).

The WHO classifies metabolic disorders as a noncommunicable chronic disease related to lifestyle and emphasizes the important role of psychological stress in its occurrence and development. Patients with depression usually fail to follow diet and weight-loss recommendations and are prone to obesity, which is a strong risk factor for metabolic diseases (Jura and Kozak, 2016). Therefore, whether behavioral changes and interventions for depression can provide benefits for the prevention of metabolic disorders is worthy of further study. This review reorganized the research progress on the management of metabolic disorder comorbidity with depression.

## Methods

All articles published before December 2020 in PubMed, Web of Science, EMBASE, Ovid, and Cochrane Library databases were searched using the terms “depression,” “metabolic disorders,” “metabolic syndrome,” and “metabolic disease.” No language restrictions were applied during the search. We also searched for relevant clinical guidelines and a list of references for related articles. Two independent researchers searched and made decisions on inclusion and exclusion criteria. When the two researchers have different opinions on the eligibility of a study for inclusion and a consensus was not reached, the senior research made the final decision after a group discussion.

Inclusion criteria were depression comorbid metabolic diseases (including but not limited to diabetes, hypertension, and hyperlipidemia), clinical studies, and literature published in English. A total of 28 literature were finally included in this study. All the keywords in the included literature were extracted, including comorbidities risk factors, comorbidities diagnostic criteria, comorbidities incidence, intervention/treatment, efficacy, and drug selection.

## Risk Factors of Metabolic Disorder Comorbidity With Depression

There are several complicated risk factors related to metabolic disorder comorbidity with depression, which is summarized as follow:

1. Demographic and behavioral factors such as age, gender, course of the disease, family history, and ethnic factors, all may be susceptible factors to metabolic disorders comorbidity with depression (Jura and Kozak, 2016; Braizat et al., 2018; Romero-Ibarguengoitia et al., 2018). In most studies, women had higher rates of depression than men (Acciai and Hardy, 2017). Among numerous sociodemographic variables, the age and disease course effect has been confirmed in several studies. The prevalence of metabolic syndrome increases with the duration of the disease and the age of patients (Jiang et al., 2018). About 30% of patients with diabetes have a family history of depression; only 3% had a family history of diabetes patients without depression (Tu et al., 2017).

Lifestyle: The increasing occurrence risk of metabolic disorders is associated with the lifestyle of patients with depression, smoking, alcohol dependence, unhealthy diet, etc. (Bly et al., 2014; Fluharty et al., 2017). All these habits also affect diseases themselves, in return, including negative symptoms and stress susceptibility. Among them, smoking is an independent risk factor for metabolic syndrome, cardiovascular disease, and type 2 diabetes (Maddatu et al., 2017; Nascimento et al., 2019). Compared with nonsmokers with depression, smokers had an increased risk of cardiovascular events in 10 years (Whooley and Wong, 2013). Also, sleep problems are common in patients with depression. About 30∼40% of depression patients have insomnia; nearly 80% of patients experience insomnia symptoms such as difficulty falling asleep and sleeping disturbance (Gebara et al., 2018). All these symptoms are significantly correlated with the occurrence of metabolic syndrome (Lin et al., 2016).

Drug factors: Antidepressants may interfere with serotonin (5-HT), the neurotransmitter that controls anxiety and mood can control appetite. These changes may increase the hunger of patients for high carbohydrates. When people are depressed, their desire will be affected. Some people would be more likely to be hungry, while others lose their appetite. These may make some people restore appetite after administration with an antidepressant, which affects their weight. Especially tricyclic anti-depressive agents (TCAs) can work on 5-HT, NE receptors, leading to increased appetite increasing and weight gain. Its direct effect on the insulin secretion of pancreatic beta cells and glucose metabolism (Deuschle, 2013) also increased the risk of metabolic syndrome in patients with depression (van Reedt Dortland et al., 2010) and accelerating the occurrence of cardiovascular disease (Serodio et al., 2014).

1. Circumstance: Under the shadow of the COVID-19 epidemics, older people and people with preexisting medical conditions (such as diabetes, heart disease, and asthma) appear to be more vulnerable to becoming severely ill with the COVID-19 virus. When COVID-19 infects people with metabolic disorders such as diabetes, treatment is more difficult because of fluctuations in blood sugar levels and potential diabetic complications. Meanwhile, COVID-19 is highly infectious, widely spread, and rapidly developing, posing a more significant threat to the physical and mental health of the public. Studies have shown that public health emergencies may cause psychological problems such as nervousness and depression in public (Shader, 2020). Therefore, changes in the environment increase the possibility of comorbid depression in patients with metabolic syndrome, and attention to the psychological state and health of older adults and people with primary diseases should not be ignored.

## Diagnosis of Metabolic Disorders Comorbidity With Depression

Metabolic syndrome requires early diagnosis, early intervention, comprehensive evaluation, and improved prognosis. Depression comorbid was speculated to aggravate the abnormal metabolism in patients with a metabolic disorder, increasing the risks of cardiovascular complications (Katon et al., 2004; Zhang et al., 2005). Clinical practice showed that the combined use of antidepressants could improve the condition in patients with metabolic disorders comorbidity with depression (Snoek et al., 2015). Accordingly, early diagnosis and intervention of depression comorbidity are critical to the success of metabolic syndrome management. However, due to the complexity of its etiology, the early diagnosis of depression in clinical practice is difficult (Smith, 2014).

At present, ICD-10 and DSM-IV are internationally accepted as classification systems for depression, while ICD-10 is the most commonly used in China. The diagnosis of a depressive episode by ICD-10 requires to have at least two core symptoms and two or more additional symptoms. Core symptoms are: black mood, lack of pleasure, and lack of energy; other symptoms are: reduced ability to focus and take the initiative, decreased self-evaluation and confidence, guilt and low self-worth, feeling the future is bleak, idea or behavior of self-injury or suicide, sleep disorders, and loss of appetite. If the symptoms continue for at least 2 weeks and do not occur secondary to alcohol or drug abuse, drug treatment, internal diseases, or bereavement reaction that can cause main daily activities or social, professional function damage, etc., should be given more attention.

Due to the complexity of the pathogenesis of the disease and the misunderstanding of the public, the clinical diagnosis rate of depression is low. Although screening tools can be used to identify patients with depression effectively, the diagnosis rate of depression among people with diabetes is still soft, and a considerable amount of depression remains undiagnosed. In patients with type 1 or type 2 adult diabetes, 1/4∼1/3 of the patients have obvious depressive symptoms but only 10∼15% have been diagnosed (Bădescu et al., 2016). As a doctor, when faced with poor glycemic and lipid control, poor treatment compliance, complaints of pain and other physical ailments frequently, and the poor doctor-patient relationship of metabolic disorders patients, the possibility of depression should be highly suspected.

Attention should be paid to the distinction between metabolic syndrome complicated with depression and psychological distress of metabolic disorders related. Professor Snoek conducted a study (Hackett et al., 2016) to distinguish patients with diabetes and concurrent depression from diabetic psychological distress. They found that depression was more complex, defined as a distinct symptom of a particular severity and duration. Depression in patients with diabetes or psychological distress caused by a chronic, self-managed illness may not be limited to such relationships. Psychological distress is the emotional response of an individual to disorders, disease management, and disease complications. Although these two psychological states exist overlap, they are not precisely synonymous. Researchers pointed out that poor treatment adherence is associated with blood glucose management outcomes, but it seems not from the same underlying cause (Golden, 2007). Routine screening for the correlation between depression and metabolic disorders, appropriate treatment, and enhanced follow-up makes significant sense for improving the quality of life of patients with metabolic syndrome and reducing medical costs (Joseph and Golden, 2017).

## Therapy Management of Metabolic Disorders Comorbidity With Depression

Patients with metabolic disease combined with depression should be monitored for the progress of metabolic symptoms and risk factors in the long-term, including blood glucose, blood lipid, diet and movement, smoking, and drug compliance. Because most metabolic disorders comorbidity with depression are a psychological burden caused by disease, the antidepressant treatment of comorbidities with metabolic disorders has an excellent effect (Fernandes et al., 2017). Diabetes is a metabolic disease that requires the most psychological and behavioral management. The American diabetes association recommends routine screening for depression in patients with diabetes, especially those with poor compliance. The treatment of metabolic syndromes complicated with depression mainly includes self-management, medication therapy, and psychological intervention.

### Self-Management and Nondrug Treatments of Comorbid Metabolic Disorders With Depression

Treatment based on the evaluation, increasing the awareness of patients about antidepression and prevention of metabolic disorders, have also been proved more feasible and effective than conventional medicine. It is recommended to evaluate and regularly monitor metabolic indicators and the degree of depressive symptoms, including body mass index (BMI), waistline, blood pressure, fasting glucose, lipid metabolism, and the score of HAMD, at the first visit or before antidepressant treatment (Hasnain et al., 2012). According to the evaluation of their metabolic risk and depression degree, patients were divided into high-risk of depression (diagnosed with metabolic disorders and depression tendency), high-risk of metabolic disease (long-term antidepressant therapy with metabolic disorder tendency), and comorbidity.

Patients with a high risk of depression should receive psychological education according to their psychological assessment status. Their metabolic indicators and psychological level should be monitored regularly. Once people with a high risk of depression have a continuously depressed and worsening mood during monitoring, or with a significantly increasing score of HAMD, approached or reached the critical standards, further and targeted intervention plans should be taken, combined with groups and individual intervention methods (Freitas et al., 2018). The intervention methods can obtain better effects, including nutrition education, cognitive behavioral therapy (CBT), cognitive therapy, interpersonal therapy, acceptance and commitment therapy, objective setting, and motivational interviewing. The complementary and alternative medicine (CAM) treatments include acupuncture, meditation, St. John’s wort, and yoga (Qaseem et al., 2016).

Adopt a multidisciplinary treatment (MDT) mode and form a team consisting of psychiatrists, nurses, and community hospitals. Conduct a continuous intervention, during hospitalization and outpatient, in the form of diet control, physical exercise, lifestyle education (regular schedule, sleep improvement, smoking cessation, and reduction of alcohol consumption), and psychological education combined with behavioral intervention. If conditions permit, nutritionists and endocrinologists can also intervene together, and long-term maintenance interventions are recommended.

**FIGURE 1:**
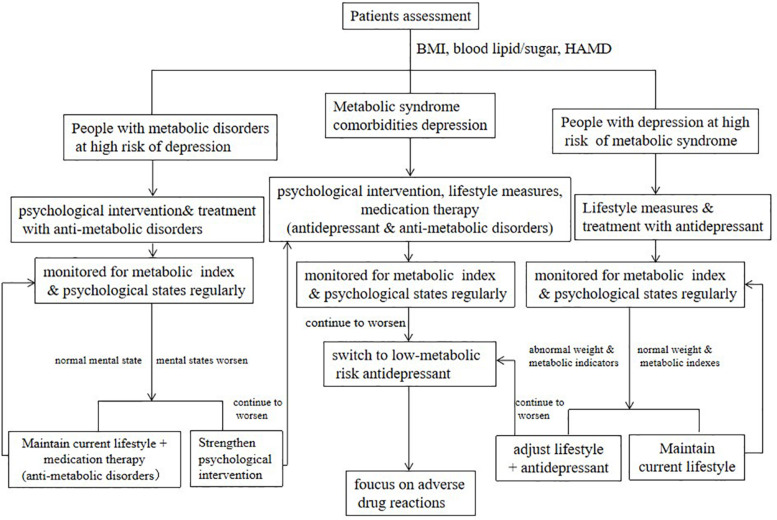
Flowchart of clinical management of metabolic disorders comorbidity with depression.

People at high risk of metabolic disorders are mainly regulated by lifestyle, including maintaining a healthy lifestyle and lifestyle intervention. The former includes a balanced diet, suitable physical exercise, and a healthy lifestyle. The content of lifestyle intervention includes alimentary control and physical activity. The general principle is to combine the metabolic problems of patients with personal preferences (such as custom, culture, religion, and economic status) to provide individualized diet management advice. Control the total daily calories while optimizing the diet and appropriately allocating the intake percentage of fats, proteins, and carbohydrates. Control sodium salt intake, increase dietary fiber intake, and supplement trace elements appropriately (Wang and Tan, 2017; Soltani et al., 2019). Cultivate an active lifestyle, increase daily activities, and reduce the duration of sedentary. Proper exercise suggestions can be given according to the physical condition and mental state of the patient, including exercise program, intensity, and frequency. Sports should be appropriate to the age, shape, and physical endurance of the patient. Lifestyle intervention of patients with metabolic disorders should be strictly implemented as a therapeutic measure. The flowchart of clinical management of metabolic disorders comorbidity with depression is shown in Figure 1.

### Medication Therapy Management of Comorbid Metabolic Disorders With Depression

The commonly used antidepressants are tricyclic antidepressants (TCA), monoamine oxidase inhibitors (MAOI), selective 5-HT reuptake inhibitors (SSRI), 5-HT, and serotonin and norepinephrine reuptake inhibitors (SNRI). TCA such as amitriptyline and imipramine, are prone to cause increased appetite, weight gain, and increased blood glucose (van Reedt Dortland et al., 2013). It is not suitable to use for people with metabolic abnormalities, especially diabetics. However, TCAs are useful, especially in patients who do not respond to other treatments. Compared with other antidepressants, MAOI often increases the sensitivity of patients to insulin and is prone to hypoglycemia, weight gain, and different adverse reactions. For example, isocarboxazid, phenelzine, and sertraline are often associated with weight loss. They are gradually being replaced by newer antidepressants, although they can improve the depressive symptoms in patients who have received other medication without effect. SSRI can reduce appetite, lower blood sugar, and is ideal for treating both diabetes and depression. However, given that such antidepressants (except citalopram) can increase blood concentrations of rosiglitazone, toluene sulfonylurea, and glipizide to varying degrees, monitoring of blood glucose concentration regularly is recommended (Fathallah et al., 2015). It is noteworthy that the increased risk of hypoglycemia after insulin administration combines with fluoxetine (Olguner Eker et al., 2017). Thus, it is not suitable to select fluoxetine. Besides, SSRIs may cause nausea and diarrhea. The side effect of weight gain often depends on the type and duration of medication. Paxil was most likely to cause weight gain, while sertraline was least likely. Venlafaxine, the SNRI representative, has a rapid onset compared with SSRI and has a significant curative effect, and is sufficient for patients with the unsatisfactory therapeutic effect of SSRI.

Neumann and Frasch (2009) found that physical activity, such as the symptoms of metabolic syndrome curative effect, especially for insulin resistance and sports activities also have a mild antidepressant effect, which proved that the hypothesis of Akbaraly is correct (Akbaraly et al., 2009). The treatment of depression is also beneficial to alleviate metabolic syndrome. A follow-up study of hospitalized patients with acute depression found that the level of triglyceride (TG) has a positive correlation with the degree of depression. A reduced TG level can be used as evidence for improving depression symptoms, suggesting that effective clinical treatment for depression can improve the metabolic levels of patients (Richter et al., 2010). After 12 weeks of treatment with citalopram in 24 patients with metabolic disorders comorbidity with depression, Poliakova et al. (2008) found that antidepressants not only can relieve depression but also can improve the metabolism of carbohydrates, lowering blood pressure and BMI. However, van Reedt Dortland et al. (2010) found that TCAs can increase the risk of patients with depression and comorbid metabolic syndrome and not depend on the severity of the depression. The reasons may be that TCAs can lead to increased abdominal fat and fat metabolism disorder. Their side effects eliminate the preventive and therapeutic effects of metabolic syndrome.

However, most of the antidepressant can affect the blood lipids, blood glucose metabolism, and even endothelial function of patients, making these complex diseases more complicated. Among antidepressants, SSRIs are the most widely used in clinical practice, and reports on their effect on lipid metabolism and endothelial function have attracted much attention.

There was a significant correlation between SSRIs concentration and lipid level, weight, and the risk of metabolic disorders (Fjukstad et al., 2016; Shi et al., 2017). A randomized controlled clinical study showed that serum levels of TG, cholesterol (TC), and low-density lipoprotein (LDL) were significantly increased in 28 patients with depression after 8 weeks of fluoxetine treatment (Pan et al., 2018). After 6 weeks of fluoxetine therapy in patients with diabetes and depression, the expression of aortic TLR4 gene and protein were significantly reduced, which reduced the levels of pro-inflammatory cytokines TNF-α and IL-1, demonstrating an improvement in metabolic, vascular, and inflammatory abnormalities in diabetes with comorbid depression (Habib et al., 2015). In a randomized controlled clinical study, after 8 weeks of sertraline combined with psychological intervention in comorbid depression of patients with diabetes, compared with the control group, fasting blood glucose and glycosylated hemoglobin were significantly decreased. Sertraline alleviated the depression of patients and effectively controlled blood glucose (Kesim et al., 2011). Comorbid depression of patients with hypertension received paroxetine combined with psychological intervention for 3 months, showing an effective relief of the depressive mood and hypertension (Humbert et al., 2019). A clinical randomized controlled study showed that during 24 weeks of treatment with escitalopram in patients with severe depression, circulating endothelial cells (CECs), vWF, and VCAM-1 was decreased, endothelial nitric oxide synthase was gradually restored to an average level, and reactive oxygen species production was reduced (Lopez-Vilchez et al., 2016). The primary antidepressant for metabolic diseases comorbid with depression and their effects on lipid metabolism and vascular endothelial function are shown in Table 1.

**TABLE 1:** The primary antidepressant for metabolic diseases comorbid with depression and their effects on metabolism.

Drugs	Diseases	Medication cycle	Study object	Therapeutic effect index	Observational index	Conclusion	Adverse
Fluoxetine	Depression	8 weeks	Clinical trial	HAMD	TG, TC, LDL, HDL	Antidepressant treatment with fluoxetine is associated with abnormal lipid metabolism	Inhibited liver enzymes, nausea, vomiting, diarrhea, dizziness, headache, insomnia, fatigue, blurred vision, constipation, tremor, anorexia
	Diabetes comorbid depression	6 weeks	Animal models	HOMA-IR*	TLR-4, TNF-α, IL-1β	Improve blood glucose and lipid in patients with diabetes comorbid depression	
Sertraline	Diabetes comorbid depression	8 weeks	Clinical trial	HAMD	Fasting blood glucose, glycosylated hemoglobin	Improve depression and control blood glucose effectively	Nausea, bellyache, indigestion, diarrhea, anorexia, tremor, dizziness, insomnia, ephidrosis, dry mouth, dysfunction
Paroxetine	Hypertension comorbid depression	3 months	Clinical trial	HAMD	Systolic and diastolic blood pressure	Improve depression and control hypertension effectively	Dry mouth, nausea, anorexia, constipation, headache, tremor, fatigue, insomnia, and sexual dysfunction
Escitalopram	Depression	24 weeks	Clinical trial	HAMD	CECs, EPC, vWF, VCAM-1	Protect vascular endothelial function	Dizziness, dysesthesia, insomnia, anxiety, nausea, vomiting, tremor, ephidrosis, headache, diarrhea, palpitations, testiness, vision blurred vision

*HOMA-IR, homeostasis model assessment-insulin resistance.

Based on lifestyle intervention and medication treatment, patients with metabolic disorders comorbidity with depression should undergo targeted treatment when abnormal metabolic indicators continue to progress and affect the treatment compliance of patients. Select or switch to drugs with low metabolic risk, and add medicines to improve metabolic problems if necessary. Switching to a low-metabolic risk antidepressant, such as sertraline, to prevent weight gain from a high-metabolic risk drug requires considering the potential for the adverse effects of multidrug therapy. Besides, joint decisions should be made with specialists to add other drugs to improve weight or metabolic abnormalities in patients with depression. The therapy function of weight gain and metabolic abnormalities of part of the oral and subcutaneous injection has been investigated in previous studies, including metformin, thiazolidinediones (TZDS), and glucagon peptide 1 receptor agonists (Ness-Abramof and Apovian, 2005). Metformin is one of the additive drugs supported by much research evidence (Ness-Abramof and Apovian, 2005; Li et al., 2019).

Most antidepressants are taken after breakfast to minimize gastrointestinal reactions. Medications that have a sleep effect may be taken at night before going to bed. If it also requires medicines for other diseases, half an hour interval is needed. Some people may suffer from constipation, nausea, vomiting, anorexia, etc. However, these are only transient reactions. Also, there may appear headaches, insomnia, sedatives, the elevation of blood pressure, etc. It is necessary to maintain medication and reexamination regularly and not abruptly withdraw without the guidance of the doctor. When adverse reactions occur, seek timely medical advice. Besides, bupropion is associated with a lower rate of sexual adverse events than fluoxetine and sertraline, whereas paroxetine has higher rates of sexual dysfunction than fluoxetine, fluvoxamine, nefazodone, and sertraline (Gartlehner et al., 2008). Physicians should discuss adverse event profiles with patients before selecting these medications.

## Conclusion

Previous studies have suggested that metabolic disorders comorbidity with depression have adverse effects on the subjective well-being and quality of life of patients, increasing the incidence and mortality of cardiovascular diseases and seriously affecting the treatment compliance of patients, requiring timely and correct management. At present, a study of patients with depression and the study of patients with metabolic syndrome, suggests that these two diseases will influence and promote each other, meanwhile, patients with metabolic syndrome are more likely to comorbidity with depression. Simultaneously, the risk of depression in patients with metabolic syndrome than the average person is high, but also study suggests they are not related. Although the co-occurrence mechanism of two diseases is not yet exact, it can be found from the pathogenesis of two conditions that they share some common disease mechanisms, such as abnormal HPA axis function and peculiar inflammatory factors. After co-occurrence of the two diseases, the treatment of an illness can prevent and treat the other condition. Using different types of antidepressants to treat complex conditions will also affect lipid metabolism and vascular endothelial cell function. Large-scale evidence-based medical research is still needed to determine the long-term efficacy safety of antidepressants in complex diseases.

Besides, comorbidity with depression with metabolic syndrome can be effectively treated by psychological and pharmaceutical means. The timely and effective treatment of depression can significantly improve the quality of life of patients with metabolic disorders, reduce the psychological burden of patients, and promote the effective development of therapy. For the first time in this study, for metabolic disorders comorbidity with depression, based on summarizing existing relevant studies and evidence, management suggestions are proposed to provide evidence for clinical diagnosis and treatment.

## Limitation

This review reorganized the research progress on the management of metabolic disorder comorbidity with depression to explore specific mechanisms for the occurrence and development of metabolic disease comorbidity with depression. It may bring a new view of reference for improving depression comorbidity metabolic diseases. However, using different types of antidepressants to treat complex conditions will also affect lipid metabolism and vascular endothelial cell function. Large-scale evidence-based medical research is still needed to determine the long-term efficacy safety of antidepressants in complex diseases.

## Author Contributions

YR conceived and designed the project. HL performed the literature retrieval and drafted the manuscript. Z-LJ provided the suggestions to improve the manuscript. All authors contributed to the article and approved the submitted version.

## Conflict of Interest

The authors declare that the research was conducted in the absence of any commercial or financial relationships that could be construed as a potential conflict of interest.

## Publisher’s Note

All claims expressed in this article are solely those of the authors and do not necessarily represent those of their affiliated organizations, or those of the publisher, the editors and the reviewers. Any product that may be evaluated in this article, or claim that may be made by its manufacturer, is not guaranteed or endorsed by the publisher.

## References

[B1] Acciai F. Hardy M. (2017). Depression in later life: a closer look at the gender gap. *Soc. Sci. Res.* 68 163–175. 10.1016/j.ssresearch.2017.08.003 29108595

[B2] Akbaraly T. N. Kivimäki M. Brunner E. J. Chandola T. Marmot M. G. Singh-Manoux A. (2009). Association between metabolic syndrome and depressive symptoms in middle-aged adults: results from the Whitehall II study. *Diab. Care* 32 499–504. 10.2337/dc08-1358 19106378 PMC2646036

[B3] Bădescu S. V. Tătaru C. Kobylinska L. Georgescu E. L. Zahiu D. M. Zăgrean A. M. (2016). The association between diabetes mellitus and depression. *J. Med. Life* 9 120–125. 27453739 PMC4863499

[B4] Bly M. J. Taylor S. F. Dalack G. Pop-Busui R. Burghardt K. J. Evans S. J. (2014). Metabolic syndrome in bipolar disorder and schizophrenia: dietary and lifestyle factors compared to the general population. *Bipolar Disord.* 16 277–288. 10.1111/bdi.12160 24330321 PMC4023536

[B5] Bot M. Pouwer F. Zuidersma M. van Melle J. P. de Jonge P. (2012). Association of coexisting diabetes and depression with mortality after myocardial infarction. *Diabetes Care* 35 503–509. 10.2337/dc11-1749 22301118 PMC3322704

[B6] Braizat O. Feinn R. Abbott G. Wagner J. (2018). Relationship style and glycaemic control in women with type 2 diabetes: the mediating role of psychological distress. *Stress Health* 34 462–467. 10.1002/smi.2795 29327498

[B7] Chisholm D. (2015). Investing in mental health. *East. Mediterr. Health J.* 21 531–534. 26442896

[B8] Dean J. Keshavan M. (2017). The neurobiology of depression: an integrated view. *Asian J. Psychiatry* 27 101–111. 10.1016/j.ajp.2017.01.025 28558878

[B9] Deuschle M. (2013). Effects of antidepressants on glucose metabolism and diabetes mellitus type 2 in adults. *Curr. Opin. Psychiatry* 26 60–65. 10.1097/YCO.0b013e32835a4206 23187087

[B10] Fathallah N. Slim R. Larif S. Hmouda H. Ben Salem C. (2015). Drug-Induced hyperglycaemia and diabetes. *Drug Saf.* 38 1153–1168. 10.1007/s40264-015-0339-z 26370106

[B11] Fernandes M. F. Mutch D. M. Leri F. (2017). The relationship between fatty acids and different depression-related brain regions, and their potential role as biomarkers of response to antidepressants. *Nutrients* 9:298. 10.3390/nu9030298 28304335 PMC5372961

[B12] Fjukstad K. K. Engum A. Lydersen S. Dieset I. Steen N. E. Andreassen O. A. (2016). Metabolic abnormalities related to treatment with selective serotonin reuptake inhibitors in patients with schizophrenia or bipolar disorder. *J. Clin. Psychopharmacol.* 36 615–620. 10.1097/jcp.0000000000000582 27749681 PMC5098465

[B13] Fluharty M. Taylor A. E. Grabski M. Munafò M. R. (2017). The association of cigarette smoking with depression and anxiety: a systematic review. *Nicotine Tob. Res.* 19 3–13. 10.1093/ntr/ntw140 27199385 PMC5157710

[B14] Freitas H. R. Isaac A. R. Malcher-Lopes R. Diaz B. L. (2018). Polyunsaturated fatty acids and endocannabinoids in health and disease. *Nutr. Neurosci.* 21 695–714. 10.1080/1028415x.2017.1347373 28686542

[B15] Gartlehner G. Gaynes B. N. Hansen R. A. Thieda P. DeVeaugh-Geiss A. Krebs E. E. (2008). Comparative benefits and harms of second-generation antidepressants: background paper for the American College of Physicians. *Ann. Intern. Med.* 149 734–750. 19017592 10.7326/0003-4819-149-10-200811180-00008

[B16] Gebara M. A. Siripong N. DiNapoli E. A. Maree R. D. Germain A. Reynolds C. F. (2018). Effect of insomnia treatments on depression: a systematic review and meta-analysis. *Depress. Anxiety* 35 717–731. 10.1002/da.22776 29782076

[B17] Golden S. H. (2007). A review of the evidence for a neuroendocrine link between stress, depression and diabetes mellitus. *Curr. Diabetes Rev.* 3 252–259. 10.2174/157339907782330021 18220683

[B18] Habib M. Shaker S. El-Gayar N. Aboul-Fotouh S. (2015). The effects of antidepressants “fluoxetine and imipramine” on vascular abnormalities and Toll like receptor-4 expression in diabetic and non-diabetic rats exposed to chronic stress. *PLoS One* 10:e0120559. 10.1371/journal.pone.0120559 25826421 PMC4380417

[B19] Hackett R. A. Kivimäki M. Kumari M. Steptoe A. (2016). Diurnal cortisol patterns, future diabetes, and impaired glucose metabolism in the whitehall II cohort study. *J. Clin. Endocrinol. Metab.* 101 619–625. 10.1210/jc.2015-2853 26647151 PMC4880118

[B20] Hasnain M. Vieweg W. V. Hollett B. (2012). Weight gain and glucose dysregulation with second-generation antipsychotics and antidepressants: a review for primary care physicians. *Postgrad. Med.* 124 154–167. 10.3810/pgm.2012.07.2577 22913904

[B21] Humbert X. Fedrizzi S. Chrétien B. Sassier M. Bagheri H. Combret S. (2019). Hypertension induced by serotonin reuptake inhibitors: analysis of two pharmacovigilance databases. *Fundam. Clin. Pharmacol.* 33 296–302. 10.1111/fcp.12440 30489655

[B22] Jiang B. Zheng Y. Chen Y. Chen Y. Li Q. Zhu C. (2018). Age and gender-specific distribution of metabolic syndrome components in East China: role of hypertriglyceridemia in the SPECT-China study. *Lipids Health Dis.* 17:92. 10.1186/s12944-018-0747-z 29678174 PMC5910574

[B23] Joseph J. J. Golden S. H. (2017). Cortisol dysregulation: the bidirectional link between stress, depression, and type 2 diabetes mellitus. *Ann. N. Y. Acad. Sci.* 1391 20–34. 10.1111/nyas.13217 27750377 PMC5334212

[B24] Jura M. Kozak L. P. (2016). Obesity and related consequences to ageing. *Age (Dordr)* 38:23. 10.1007/s11357-016-9884-9883 26846415 PMC5005878

[B25] Katon W. J. Lin E. H. Russo J. Von Korff M. Ciechanowski P. Simon G. (2004). Cardiac risk factors in patients with diabetes mellitus and major depression. *J. Gen. Intern. Med.* 19 1192–1199. 10.1111/j.1525-1497.2004.30405.x 15610329 PMC1492592

[B26] Kesim M. Tiryaki A. Kadioglu M. Muci E. Kalyoncu N. I. Yaris E. (2011). The effects of sertraline on blood lipids, glucose, insulin and HBA1C levels: a prospective clinical trial on depressive patients. *J Res Med Sci* 16 1525–1531. 22973359 PMC3434892

[B27] Kupferberg A. Bicks L. Hasler G. (2016). Social functioning in major depressive disorder. *Neurosci. Biobehav. Rev.* 69 313–332. 10.1016/j.neubiorev.2016.07.002 27395342

[B28] Li G. F. Zhao M. Zhao T. Cheng X. Fan M. Zhu L. L. (2019). Effects of metformin on depressive behavior in chronic stress rats. *Zhongguo Ying Yong Sheng Li Xue Za Zhi* 35 245–249. 10.12047/j.cjap.5775.2019.052 31257807

[B29] Li H. Luo X. Ke X. Dai Q. Zheng W. Zhang C. (2017). Major depressive disorder and suicide risk among adult outpatients at several general hospitals in a Chinese Han population. *PLoS One* 12:e0186143. 10.1371/journal.pone.0186143 29016669 PMC5634639

[B30] Lin S. C. Sun C. A. You S. L. Hwang L. C. Liang C. Y. Yang T. (2016). The link of self-reported insomnia symptoms and sleep duration with metabolic syndrome: a chinese population-based study. *Sleep* 39 1261–1266. 10.5665/sleep.5848 27070137 PMC4863215

[B31] Lopez-Vilchez I. Diaz-Ricart M. Navarro V. Torramade S. Zamorano-Leon J. Lopez-Farre A. (2016). Endothelial damage in major depression patients is modulated by SSRI treatment, as demonstrated by circulating biomarkers and an in vitro cell model. *Transl. Psychiatry* 6:e886. 10.1038/tp.2016.156 27598970 PMC5048198

[B32] Maddatu J. Anderson-Baucum E. Evans-Molina C. (2017). Smoking and the risk of type 2 diabetes. *Transl. Res.* 184 101–107. 10.1016/j.trsl.2017.02.004 28336465 PMC5429867

[B33] Nascimento G. G. Leite F. R. M. Peres K. G. Demarco F. F. Corrêa M. B. Peres M. A. (2019). Metabolic syndrome and periodontitis: a structural equation modeling approach. *J. Periodontol.* 90 655–662. 10.1002/jper.18-0483 30447085

[B34] Ness-Abramof R. Apovian C. M. (2005). Drug-induced weight gain. *Drugs Today (Barc)* 41 547–555. 10.1358/dot.2005.41.8.893630 16234878

[B35] Neumann N. U. Frasch K. (2009). Coherences between the metabolic syndrome, depression, stress and physical activity. *Psychiatry Prax* 36 110–114. 10.1055/s-2008-1067558 18924063

[B36] Olguner Eker Ö Özsoy S. Eker B. Doğan H. (2017). Metabolic effects of antidepressant treatment. *Noro Psikiyatr Ars* 54 49–56. 10.5152/npa.2016.12373 28566959 PMC5439472

[B37] Pan S. J. Tan Y. L. Yao S. W. Xin Y. Yang X. Liu J. (2018). Fluoxetine induces lipid metabolism abnormalities by acting on the liver in patients and mice with depression. *Acta Pharmacol. Sin.* 39 1463–1472. 10.1038/aps.2017.207 30150788 PMC6289401

[B38] Poliakova E. O. Shimchik V. E. Mychka V. B. Chazova I. E. (2008). The role of psychopharmacotherapy in combined treatment of patients with metabolic syndrome and depression. *Ter Arkh* 80 69–73. 18491585

[B39] Qaseem A. Barry M. J. Kansagara D. (2016). Nonpharmacologic versus pharmacologic treatment of adult patients with major depressive disorder: a clinical practice guideline from the american college of physicians. *Ann. Intern. Med.* 164:350. 10.7326/m15-2570 26857948

[B40] Richter N. Juckel G. Assion H. J. (2010). Metabolic syndrome: a follow-up study of acute depressive inpatients. *Eur. Arch. Psychiatry Clin. Neurosci.* 260 41–49. 10.1007/s00406-009-0013-15 19399357

[B41] Romero-Ibarguengoitia M. E. Vadillo-Ortega F. Caballero A. E. Ibarra-González I. Herrera-Rosas A. Serratos-Canales M. F. (2018). Family history and obesity in youth, their effect on acylcarnitine/aminoacids metabolomics and non-alcoholic fatty liver disease (NAFLD). *Struct. Equat. Model. Approach* 13:e0193138. 10.1371/journal.pone.0193138 29466466 PMC5821462

[B42] Saklayen M. G. (2018). The global epidemic of the metabolic syndrome. *Curr. Hypertens. Rep.* 20:12. 10.1007/s11906-018-0812-z 29480368 PMC5866840

[B43] Serodio K. J. Ardern C. I. Rotondi M. A. Kuk J. L. (2014). Tricyclic and SSRI usage influences the association between BMI and health risk factors. *Clin. Obes* 4 296–302. 10.1111/cob.12067 25826158

[B44] Shader R. I. (2020). COVID-19 and depression. *Clin. Ther.* 42 962–963. 10.1016/j.clinthera.2020.04.010 32362345 PMC7184005

[B45] Shi Z. Atlantis E. Taylor A. W. Gill T. K. Price K. Appleton S. (2017). SSRI antidepressant use potentiates weight gain in the context of unhealthy lifestyles: results from a 4-year Australian follow-up study. *BMJ Open* 7:e016224. 10.1136/bmjopen-2017-016224 28801419 PMC5629701

[B46] Smith K. (2014). Mental health: a world of depression. *Nature* 515:181. 10.1038/515180a 25391942

[B47] Snoek F. J. Bremmer M. A. Hermanns N. (2015). Constructs of depression and distress in diabetes: time for an appraisal. *Lancet Diab. Endocrinol.* 3 450–460. 10.1016/s2213-8587(15)00135-137 25995123

[B48] Soltani S. Kolahdouz Mohammadi R. Shab-Bidar S. Vafa M. Salehi-Abargouei A. (2019). Sodium status and the metabolic syndrome: a systematic review and meta-analysis of observational studies. *Crit. Rev. Food Sci. Nutr.* 59 196–206. 10.1080/10408398.2017.1363710 28846446

[B49] Tao R. Li H. (2015). High serum uric acid level in adolescent depressive patients. *J. Affect. Disord.* 174 464–466. 10.1016/j.jad.2014.12.031 25553407

[B50] Tu H. P. Hsieh H. M. Liu T. L. Jiang H. J. Wang P. W. Huang C. J. (2017). Prevalence of depressive disorder in persons with type 2 diabetes: a national population-based cohort study 2000-2010. *Psychosomatics* 58 151–163. 10.1016/j.psym.2016.11.007 28190545

[B51] van Reedt Dortland A. K. Giltay E. J. van Veen T. Zitman F. G. Penninx B. W. (2010). Metabolic syndrome abnormalities are associated with severity of anxiety and depression and with tricyclic antidepressant use. *Acta Psychiatr. Scand.* 122 30–39. 10.1111/j.1600-0447.2010.01565.x 20456284

[B52] van Reedt Dortland A. K. Vreeburg S. A. Giltay E. J. Licht C. M. Vogelzangs N. van Veen T. (2013). The impact of stress systems and lifestyle on dyslipidemia and obesity in anxiety and depression. *Psychoneuroendocrinology* 38 209–218. 10.1016/j.psyneuen.2012.05.017 22717171

[B53] Wang N. Tan H. Y. (2017). Supplementation of micronutrient selenium in metabolic diseases: its role as an antioxidant. *Oxid. Med. Cell Longev.* 2017:7478523. 10.1155/2017/7478523 29441149 PMC5758946

[B54] Whooley M. A. Wong J. M. (2013). Depression and cardiovascular disorders. *Annu. Rev. Clin. Psychol.* 9 327–354. 10.1146/annurev-clinpsy-050212-185526 23537487

[B55] Yu M. Zhang X. Lu F. Fang L. (2015). Depression and risk for diabetes: a meta-analysis. *Can. J. Diabetes* 39 266–272. 10.1016/j.jcjd.2014.11.006 25773933

[B56] Zhang X. Norris S. L. Gregg E. W. Cheng Y. J. Beckles G. Kahn H. S. (2005). Depressive symptoms and mortality among persons with and without diabetes. *Am. J. Epidemiol.* 161 652–660. 10.1093/aje/kwi089 15781954

